# Correlation between single limb support phase and self-evaluation questionnaires in knee osteoarthritis populations

**DOI:** 10.3109/09638288.2010.520805

**Published:** 2011-01-05

**Authors:** RONEN DEBI, AMIT MOR, GANIT SEGAL, OFER SEGAL, GABRIEL AGAR, EYTAN DEBBI, NAHUM HALPERIN, AMIR HAIM, AVI ELBAZ

**Affiliations:** 1 Department of Orthopedic Surgery, Assaf Harofeh Medical Center, Zerifin, Israel; 2 AposTherapy Research Group, Herzliya, Israel; 3 Sourasky Medical Center, Tel Aviv, Israel

**Keywords:** Single limb support, osteoarthritis, gait, WOMAC, SF-36

## Abstract

*Purpose.* To investigate the correlation between single limb support (SLS) phase (% of gait cycle) and the Western Ontario and McMaster University Osteoarthritis Index (WOMAC) questionnaire and Medical Outcomes Study 36-Item Short-Form Health Survey (SF-36 Health Survey) in patients with knee osteoarthritis (OA).

*Method.* A prospective observational study was employed with 125 adults with bilateral medial compartment symptomatic knee OA who underwent a physical and radiographic evaluation. Velocity, step length and SLS were assessed by a computerised mat (GAITRite). Patients completed the WOMAC and SF-36 Health Survey questionnaires. *Results.* Statistical analysis examined the correlations between SLS and both questionnaires, between Kellgren & Lawrence (K&L) scores and both questionnaires and between SLS correlations and K&L correlations. We found significantly stronger correlations between SLS and WOMAC-pain, WOMAC-function, the SF-36 pain sub-category, velocity and step length than between K&L scores and these parameters (Pearson's *r—* 0.50 *vs.* 0.26, 0.53 *vs.* 0.34, 0.50 *vs.* 023, 0.81 *vs.* 0.33, 0.77 *vs.* 0.37, respectively; *all p 5* 0.05). Significant differences in SLS were found over WOMAC-pain, WOMAC-function and SF-36 overall score quartiles *(p 5* 0.05 for all).

*Conclusion.* We recommend integrating SLS as an objective parameter in the comprehensive evaluation of patients with knee OA.

## Introduction

In order to understand and assess the symptoms and functional severity of patients suffering from knee osteoarthritis (OA), clinicians and researchers use validated self-evaluation questionnaires such as the Western Ontario and McMaster Universities Osteoarthritis Index (WOMAC) and the Medical Outcomes Study 36-Item Short-Form Health Survey (SF-36 Health Survey) [1,2]. Nevertheless, there is a lack in accurate, objective, non-invasive and simple clinical tools for assessing knee OA in terms of functional independence and performance.

In recent years, researchers have gained an increased understanding of important biomechanical gait pattern changes that occur during the pathogen-esis and progression of knee OA [3-5]. Studies comparing the gait spatio-temporal parameters of patients with knee OA with those of healthy individuals have shown that patients with knee OA tend to have a slower walking speed, shorter step length and shorter single limb support (SLS) [4,6,7].

The SLS value is a percent of the gait cycle that corresponds to the time spent on one limb while the contralateral limb swings forward. In healthy populations, this phase accounts for 38-40% of the gait cycle [8]. While a relationship has yet to be clearly established between decreased loads due to pain and decreased SLS values, studies have shown that patients with knee OA attempt to avoid pain by decreasing loads from the affected joint [9]. A patient can achieve this by decreasing the SLS phase and increasing the double limb support (DLS) phase. A valid assumption is, therefore, that patients with knee OA demonstrate lower SLS values because they are trying to alleviate their pain. The same may be true for walking speed and stride length. We have shown in a previous study, however, that while walking speed is a parameter that can be consciously controlled by the patient, the SLS is less sensitive [10].

In order to confirm whether this is a valid and feasible parameter for clinical assessment of knee OA, SLS must first be compared to the gold standard questionnaires currently being used for evaluating knee OA. The purpose of this study was to examine the correlation between the SLS phase and the WOMAC questionnaire and SF-36 Health Survey.

## Methods

### Study participants

This was a prospective observational study on 125 patients with bilateral medial compartment knee OA. Patients were recruited to the study from the Orthopaedic Outpatient Clinic at Assaf Harofeh Medical Centre in Zerifin, Israel during their routine quarterly examination. Patients were also recruited from AposTherapy Centre in Herzliya, Israel during their first visit to the clinic. Patients arrived for the first visit at the hospital or clinic due to complaints of knee pain. The protocol was approved by Assaf Harofeh Medical Centre Institutional Helsinki Committee Registry (Helsinki registration number 185/07, NIH protocol no. NCT00599729). All patients volunteered and gave written informed consent before entering the study.

Eligibility was defined as having symptomatic knee OA for at least 6 months, fulfilling the ACR clinical criteria for OA of the knee [11], and having radio-graphically assessed OA of the knee according to the K&L scale [12]. Exclusion criteria were acute septic arthritis, inflammatory arthritis and corticosteroid injection within 3 months of the study, avascular necrosis, history of knee buckling or recent knee injury, joint replacement, neuropathic arthropathy, increased tendency to fall due to neurological disorders, lack of physical or mental ability to perform or comply with the study procedure, and history of pathological osteoporotic fracture. Patients were examined by an orthopaedic surgeon at each site to determine eligibility.

### Protocol

All patients were radiographically assessed based on their most recently available radiograph by the senior author (N.H.) using the K&L scale. The senior author was blinded to the data collection process (gait analysis and questionnaires). The radiographs were obtained using a standardised technique [13]. Briefly, the images were 458 posteroanterior flexion weight-bearing radiographs. Patients were instructed to flex both knees to 458 and make an effort to distribute their weight evenly on the two extremities. Toes were pointed straight ahead and the patellae touched the film cassette. The radiographic machine was positioned 101.6 cm away from the cassette. While all patients had lateral view radiographs, the K&L scoring was conducted on the tibio-femoral joint since this was the most symptomatic area in all patients. In addition, patients underwent a physical examination and anthropometric measurements of height, weight and leg length (measured from the tip of the greater trochanter to the floor through the lateral melleolus in an upright standing position) [14]. The gait spatio-temporal parameters were measured with a computerised walkway system (GAITRite, CIR Systems Inc.). The computerised system is an electronic walkway mat 4.87 m long. The spatio-temporal parameters are measured, processed and stored on a PC computer running the GAITRite Platinum software (version 3.9). Patients were instructed to walk barefoot at a self-selected speed since previous studies have shown that this speed gives consistent gait parameters results [15,16]. Patients were instructed to walk 3 m before and after the walkway mat to allow sufficient acceleration and deceleration time outside the measurement area. Each gait test included six walks and the mean value of the six walks was calculated for each parameter. Following the gait test patients completed the WOMAC questionnaire and the SF-36 Health Survey. Since the gait test only takes 3 min to complete it is reasonable to assume that no fatigue will occur that could affect the results of the questionnaires.

A valid Hebrew translation of the WOMAC Index VAS scale questionnaire was used (version 3.1) [17]. Results range from 0 cm-10 cm, where 0 indicates no pain, stiffness and functional limitation and 10 indicates severe pain, stiffness and functional limitation. A valid Hebrew translation of the SF-36 original Likert scale was used [18]. The results range between 0 and 100, where 0 indicates poor quality of life and 100 indicates excellent quality of life.

The following parameters were evaluated: radio-graphic assessment according to K&L grading scale, SLS (% gait cycle), gait velocity (cm/s), step length (cm), WOMAC-pain, WOMAC-function and quality of life using the SF-36 Health Survey sub-categories.

### Statistical analysis

For each patient, the leg with the lowest mean SLS value was chosen for comparison to the questionnaire data and this value was used for all analyses. SLS represents the phase in the gait cycle when the body weight is entirely supported by one limb while the contralateral limb swings forward. Therefore, we assumed that the knee with the lowest SLS value would have greater difficulty bearing loads. The K&L data for this same leg were also used for comparison to the questionnaire data. Data were analysed using SPSS statistic software (version 17.0). The distributions of gait characteristics were examined using the Kolmogorov-Smirnov non-parametric test. Descriptive statistics were calculated for all continuous parameters. Pearson correlations and 95% confidence intervals for the correlation coefficients were calculated in order to demonstrate the correlation between WOMAC-pain, WOMAC-function and SF-36 scales and measures of knee OA severity (K&L and SLS). Values of *r*≤0.25 were considered low, values of 0.25 <*r*≤ 0.50 were considered moderate and values of *r*≥0.75 were considered high [19]. Comparisons between the correlation coefficients of the questionnaires (WOMAC-pain, WOMAC-function and SF-36 scales) to SLS and to K&L were conducted according to Meng et al. [20]. SLS means were compared over quartiles of the WOMAC scores using the one way analysis of variance (ANOVA) test. Differences in SLS medians were demonstrated in box plots. All statistical tests were two-sided. The level of significance for all statistical tests was set at *p* < 0.05.

## Results

Patient characteristics are presented in Table I. Gait velocity and step length were normalised to leg length in order to eliminate the effect of leg length on these parameters [21]. While preferable in analysis, these normalised values did not affect the correlation results. Results of the spatio-temporal parameters and the questionnaires scores are summarised in Tables II and III, respectively.

**Table I:** Baseline patient characteristics (*n* = 125).

	Total			
	Frequency	Mean	SD	Percentage (%)
Male gender	46			37
Age (years)		65.9	11.2	
Height (cm)		160	8.7	
Weight (kg)		82.1	15.8	
Body mass index (BMI) (kg/m^2^)		31.9	5.9	
Left leg length (cm)		83.0	5.9	
Right leg length (cm)		82.9	5.8	
K&L* Scale Grade 1	26			20.8
K&L Scale Grade 2	32			25.6
K&L Scale Grade 3	35			28
K&L Scale Grade 4	32			25.6

* Kellgren & Lawrence.

**Table II:** Spatio-temporal parameters measured by gait analysis.

Parameter	Minimum	Maximum	Mean (SD)
Velocity (cm/s)	25.8	147.9	94.3 (23.0)
Normalised velocity (cm/s/leg length)	0.33	1.73	1.13 (0.3)
Cadence (steps/min)	65.8	131.3	105.3 (12.6)
Step length left (cm)	21.1	77.3	52.9 (9.3)
Step length right (cm)	19.1	76.2	53.4 (9.3)
Normalised step length left (cm/leg length)	0.27	0.86	0.63 (0.1)
Normalised step length right (cm/leg length)	0.25	0.88	0.64 (0.1)
Single limb support left (% gait cycle)	24.0	42.4	36.5 (3.3)
Single limb support right (% gait cycle)	25.0	42.9	36.9 (3.1)

**Table III:** WOMAC osteoarthritis index and SF-36 health survey scores.

Parameter	Minimum	Maximum	Mean (SD)
WOMAC			
Pain	0	9.6	4.4 (2.6)
Function	0	9.6	4.4 (2.7)
SF-36			
Physical functioning	0	100	46.4 (25.4)
Role limitation due to physical health	0	100	47 (40.6)
Role limitation due to emotional problems	0	100	63.9 (41)
Energy/fatigue	0	100	55.4 (22.0)
Emotional well being	12	100	70.3 (18.3)
Social functioning	0	100	73.5 (25.7)
Pain	0	100	46.3 (26.3)
General health	12.5	91.7	58.6 (16.9)

The correlations between the K&L score and WOMAC-pain, WOMAC-function and SF-36 sub-categories were low to moderate, while the correlations between the SLS and the questionnaires were moderate. The correlations between SLS and the questionnaires were significantly stronger than the correlations between the K&L and the questionnaires. High correlations were also found between SLS and both normalised velocity and normalised step length. All correlation results are summarised in Table IV.

**Table IV:** Correlation between the WOMAC-pain, WOMAC-function, SF-36 sub-categories, normalised velocity, normalised step length parameters and both K&L scale and SLS.

Parameter	K&L† Grading Scale (0-4), *r*‡	95% CI	Single limb support, *r*‡	95% CI	p*
WOMAC§					
Pain	0.26	0.07-0.4	−0.50	−0.62 to −0.35	0.01
Function	0.34	0.17-0.48	−0.53	−0.63 to −0.37	0.03
SF-36¶					
Physical functioning	−0.39	−0.53 to −0.23	0.49	0.34-0.61	0.25
Energy/fatigue	−0.15**	−0.32 to-0.03	0.33	0.16-0.48	0.06
Pain	−0.23	−0.39 to −0.06	0.50	0.36-0.62	≤0.01
General health	−0.21	−0.37 to −0.04	0.36	0.20-0.50	0.12
Normalised velocity	−0.33	−0.48 to −0.16	0.81	0.74-0.86	<0.01
Normalised step length	−0.37	−0.51 to −0.21	0.77	0.69-0.83	<0.01

* *p* values refer to significant differences between Pearson correlation coefficients of the questionnaires to SLS and to K&L according to the method of Meng et al.

** Not statistically significant (*p* = 0.154).

† Kellgren and Lawrence.

‡ *r* = Pearson correlation coefficient.

§ Western Ontario and McMaster University Osteoarthritis Index.

¶ Medical outcomes study 36-item short-form health survey.

**Figure 1:**
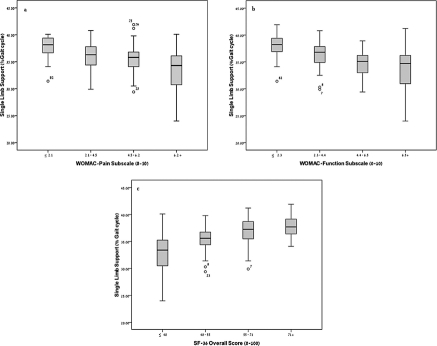
Single limb support distribution according to (a) WOMAC-pain, (b) WOMAC function and (c) SF-36 overall score quartiles. The box plots represent the median value of SLS with the range of the 1st quartile and the 3rd quartile.

We further investigated the mean SLS value in WOMAC-pain, WOMAC-function and SF-36 overall score (Tables V-VII). It can be seen that as the level of pain and functional limitation increases, the mean SLS decreases (all *p 5* 0.05). Additionally, as the quality of life increases (SF-36 overall score), the mean SLS increases *(p 5* 0.05). This relationship is further illustrated in the box plots showing the median SLS in WOMAC-pain (Figure 1a), WOMAC-function (Figure 1b) and SF-36 overall score quartiles (Figure 1c). This distribution in the quartile categories further elucidates the correlations reported above.

**Table V:** Distribution of mean SLS values over WOMAC-pain quartiles.

	WOMAC*-pain (cm) quartiles			
	Q1	Q2	Q3	Q4
	≤2.1	2.1–4.5	4.5–6.2	≥6.2
SLS† (%)‡	37.7	36.1	35.5	33.4
95% C.I.	37.0–38.5	35.1–37.1	34.5–36.5	32.0–34.7

* Western Ontario and McMaster University Osteoarthritis Index.

† Single limb support.

‡ One-way ANOVA test. Significance level was set at *p* < 0.05. Significant differences in SLS were found over the quartiles.

**Table VI:** Distribution of mean SLS values over WOMAC-function quartiles.

	WOMAC*-function (cm) quartiles			
	Q1	Q2	Q3	Q4
	≤2.3	2.3–4.4	4.4–6.5	≥6.5
SLS† (%)‡	37.9	36.4	34.6	33.8
95% C.I.	37.2–38.7	35.5–37.3	33.7–35.4	32.3–35.2

† Western Ontario and McMaster University Osteoarthritis Index.

‡ Single limb support. ‡One-way ANOVA test. Significance level was set at *p* < 0.05.

Significant differences in SLS were found over the quartiles.

**Table VII:** Distribution of mean SLS values over SF-36 overall score quartiles.

	SF-36* Overall Score quartiles			
	Q1	Q2	Q3	Q4
	≤40	40–55	55–71	≥71
SLS† (%)‡	32.9	35.4	36.8	37.7
95% C.I.	31.6–34.2	34.4–36.3	35.8–37.8	37.0–38.4

* Medical outcomes study 36-item short-form health survey.

† Single limb support.

‡ One-way ANOVA test. Significance level was set at *p* < 0.05. Significant differences in SLS were found over the quartiles.

## Discussion

The purpose of this study was to examine the correlation of an objective gait parameter with the level of pain and function and with the quality of life perception of patients suffering from knee OA. Since the ability of the radiographic assessment to reflect the functional and dynamic condition of patients with knee OA is limited, we found it important to add an objective, non-invasive parameter that will help evaluate the functional severity of knee OA.

The gait pattern differences between healthy individuals and patients with knee OA illustrate the impact of the disease on mobility parameters [6]. Specifically, Brandes et al. reported lower SLS values in both limbs among patients with knee OA compared to SLS values of healthy individuals [7].

We assumed that patients with severe pain and functional limitation will have lower SLS values, whereas patients with minimal pain and functional limitation will demonstrate higher SLS values. The current study found moderate correlations between the SLS parameter and the WOMAC-pain, WO-MAC-function and the sub-categories of the SF-36 Health Survey questionnaires. When examining the correlation between two independent variables (SLS and WOMAC-pain) that reflect OA severity from a different perspective it is expected that a moderate correlation can be considered to be a good correlation. In contrast, the correlation between two identical measurements (i.e. two different blood pressure gauges) that measure the same parameter would be expected to be much higher.

These results indicate that SLS can express the level of pain and functional limitation of patients with knee OA and may also reflect a patient's functional condition during different daily tasks. SLS may, therefore, be a helpful tool for examining knee OA functional severity clinically. Evaluations of structural severity, however, such as a K&L assessment, are still important in examining knee OA. Future studies should further examine the role of SLS as a clinical objective indicator for the severity of knee OA.

Previous studies have reported that patients with knee OA walk slower and have a shorter step length compared to healthy age-matched individuals [6,7]. It may, therefore, seem that simple gait tests monitoring gait velocity, such as ‘time up and go’ tests, are enough to evaluate a patient's functional ability. But as with self-evaluation questionnaires, such tests do not have independency since patients can consciously control their gait velocity and step length. SLS on the other hand is less sensitive to changes and is probably more difficult for the patient to voluntarily control [10].

This study had limitations with regard to the study population. Only patients with clinical and radio-graphic knee OA were included in this study [11]. It would have been preferable to recruit patients experiencing knee pain and functional limitations that do not have radiographic evidence of knee OA. Examining the correlations between SLS and pain, function and quality of life in patients reporting knee pain without radiographic symptoms might help in identifying early stages of knee OA disease that are not yet reflected in radiographic evaluation. For this reason our analysis of SLS as a functional severity assessment tool is limited only to patients having structural OA deformities.

The findings of this study suggest that inclusion of an objective measurement tool such as spatio-temporal parameters and particularly SLS can help in evaluating knee OA severity, effectiveness of treatment and might even help in disease management. Furthermore, SLS may serve as a simple follow-up measurement in patients already diagnosed with and in those being treated for knee OA and other disorders. A medical practitioner may, therefore, find it beneficial to send patients for inexpensive gait.

## Conclusions

SLS correlated with WOMAC-pain, WOMAC-function, SF-36, velocity and step length. We found stronger correlations between SLS and WOMAC-pain, WOMAC-function and SF-36 pain than between K&L scores and these parameters. Therefore, we recommend integrating the SLS as an objective parameter in the comprehensive evaluation of a patient with knee OA. Further investigation is needed regarding the use of SLS as a clinical indicator for the functional severity of knee OA.
